# A first report of the detection of *Avipoxvirus* genomic sequences in louse flies (Diptera: Hippoboscidae)

**DOI:** 10.1017/S0031182025000526

**Published:** 2025-04

**Authors:** Denise Wawman, Ben P. Jones, Steven R. Fiddaman, Jane E. Turner, Nicholas Johnson, Adrian L. Smith

**Affiliations:** 1 Edward Grey Institute of Field Ornithology, Department of Biology, University of Oxford, Oxford, UK; 2 Vector-Borne Disease Workgroup, Virology Department, Animal and Plant Health Agency, Addlestone, UK; 3 Department of Biology, University of Oxford, Oxford, UK; 4 Independent Researcher, Wirral Peninsula, UK; 5 Faculty of Health and Medical Sciences, University of Surrey, Guildford, SRY, UK

**Keywords:** avian pox, disease detection, methods, parasite, vector, wildlife disease

## Abstract

The Hippoboscidae are ectoparasites of birds and mammals, which, as a group, are known to vector multiple diseases. *Avipoxvirus* (APV) is mechanically vectored by various arthropods and causes seasonal disease in wild birds in the United Kingdom (UK). Signs of APV and the presence of louse flies (Hippoboscidae) on Dunnocks *Prunella modularis* were recorded over a 16·5-year period in a rural garden in Somerset, UK. Louse flies collected from this site and other sites in England were tested for the presence of APV DNA and RNA sequences. Louse flies on Dunnocks were seen to peak seasonally three weeks prior to the peak of APV lesions, an interval consistent with the previously estimated incubation period of APV in Dunnocks. APV DNA was detected on 13/25 louse flies, *Ornithomya avicularia* and *Ornithomya fringillina*, taken from Dunnocks, both with and without lesions consistent with APV, at multiple sites in England. Collectively these data support the premise that louse flies may vector APV. The detection of APV in louse flies, from apparently healthy birds, and from sites where disease has not been observed in any host species, suggests that the Hippoboscidae could provide a non-invasive and relatively cheap method of monitoring avian diseases. This could provide advanced warnings of disease, including zoonoses, before they become clinically apparent.

## Graphical abstract

**Figure:**
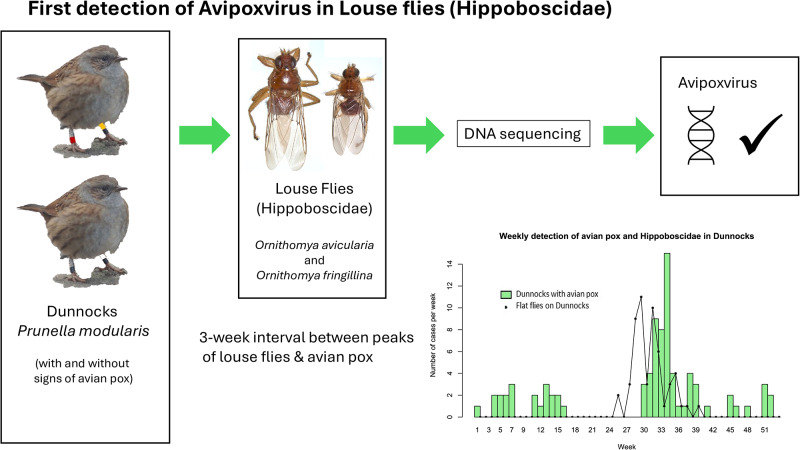

## Introduction

The Diptera in the family Hippoboscidae are related to the Tsetse flies, and both sexes are obligate haematophagous ectoparasites. There are over 200 species worldwide (Dick, 2018) of which 11 species breed in the United Kingdom (UK), 3 on mammals (keds) and 8, louse or flat flies, on birds (Hutson, 1984; Wawman, 2024).

Some species of louse fly are proven vectors of various pathogens and others have been isolated from them without formal proof that these flies are acting as vectors (Bezerra-Santos and Otranto, 2020). *Ornithomya avicularia* and *Pseudolynchia canariensis* are proven biological vectors of *Haemoproteus* sp. (Baker, 1963; Cepeda et al., 2019), and trypanosomes have been shown to develop within the midgut of *O. avicularia*, and to reach the infective stage, but transmission only occurs when birds ingest the flies (Baker, 1967). For most other pathogens, the evidence that louse flies are acting as vectors is weaker: West Nile Virus (WNV) was detected in *Icosta americana* from WNV infected raptors (Farajollahi et al., 2005); *Crataerina melbae* was proposed as a vector of trypanosomes in Alpine Swifts *Tachymarptis melba* (Cigler et al., 2024); an unidentified Hippoboscid was suggested as a potential vector when found in association with Newcastle Disease Virus infected Laughing Doves *Streptopelia senegalensis* (Obanda et al., 2016). Pathogens have also been identified in louse flies taken from birds without documented signs of disease, including *Babesia* spp. from *O. avicularia* and *O. biloba* (Čisovská Bazsalovicsová et al., 2023), *Rickettsia* sp. from *Crataerina pallida* (Cerutti et al., 2018), and trypanosomes from *O. biloba, O. fringillina* and *Ornithoica turdi* (Santolíková et al., 2022).

*Avipoxvirus* (APV) is a genus of double-stranded DNA viruses within the Chordopoxvirinae subfamily and Poxviridae family (ICTV, 2024). The most studied member of the genus is fowlpox virus, a cause of disease in domestic poultry (Van Riper and Forrester, 2007; Yeo et al., 2019). Other members of the genus cause disease in wild avian species, which is characterised by proliferative lesions on the skin, feet and legs, head and sometimes on the mucus membranes. APV is mechanically vectored by a range of arthropods, including *Aedes* and *Culex* mosquitoes (Kligler et al., 1929), midges (Lee et al., 2017), fleas (Smits et al., 2005), the mite *Dermanyssus gallinae* (Shirinov et al., 1972; Huong et al., 2014) and Stable Flies *Stomoxys calcitrans* (Alehegn et al., 2014). APV remains viable on the proboscis of *Culex tarsalis* for up to 28 days (DaMassa, 1966). In temperate regions of the world APV is a seasonal disease where vectors are not active during the winter (Wawman *et al.*, unpublished results; Van Riper and Forrester, 2007; Lachish et al., 2012).

The Dunnock *Prunella modularis* is a small passerine that is native across Europe. It was first reported with APV lesions in the UK in the 1940’s (Edwards, 1955). It is affected by APV with a seasonal peak in August following an absence of disease in May and June (Wawman *et al.*, unpublished results) which correlates with the seasonal peaks in vectors such as mosquitoes (Cull et al., 2024). Three species of Hippoboscid have been found on UK Dunnocks, *O. avicularia, O. fringillina* and *O. chloropus* (Wawman, Smith and Sheldon, unpublished results; Hill, 1962) and their peaks in populations occur seasonally at the time of year which might potentially allow them to be vectors of APV in Dunnocks (Wawman, 2025). This is particularly true when the estimated incubation period, which varies from 13 to 48 days in Dunnocks (Wawman *et al.*, unpublished results), is taken into consideration.

In this study we combine knowledge of the seasonality of APV and Hippoboscids on Dunnocks, with molecular techniques to detect APV in association with *Ornithomya* spp. We also consider whether louse flies and keds could be used as a non-invasive way to sample for viruses in wild populations.

## Materials and methods

### Samples

From February 2008 until August 2024, Dunnocks were ringed, in a rural garden, near Minehead, in Somerset, as part of the British Trust for Ornithology (BTO) Retrapping Adults for Survival Scheme (https://www.bto.org/our-science/publications/birdtrends/2020/methods/retrapping-adults-survival-scheme, last accessed 9 September 2024). At each capture, in addition to the usual data collected during bird ringing, records were made of signs of disease or the presence of ectoparasites. Louse flies that left Dunnocks during routine ringing activities were collected, identified according to a key (Hutson, 1984), and preserved in 70% ethanol (with distilled water and no other excipients).

During 2023, both at the main APV study site near Minehead in Somerset, and at a second site on the Wirral Peninsula near Liverpool where lesions consistent with APV had been observed, flies were preserved in RNAlater for DNA and RNA analyses.

Additional flies were collected from all bird species from 2020 to 2023, as part of the ‘Mapping the UK’s Flat Flies Project’ (Wawman, 2025) and stored in 70% ethanol for later analyses.

### Next generation sequencing (NGS)

One fly, taken from a Dunnock with APV at the second site, and the only one from an infected Dunnock that was preserved in RNAlater, was chosen for DNA and RNA extraction, using shotgun Next Generation Illumina Sequencing (NGS). The fly was prepared by washing in phosphate buffered saline (PBS) to remove the RNAlater, then in 5% sodium hypochlorite solution to remove contaminants, and finally twice more in PBS to remove the sodium hypochlorite.

DNA and RNA were isolated separately from the fly using the QIAgen AllPrep DNA/RNA MiniKit (Qiagen, Manchester, UK). Briefly, the fly was homogenized in 300µl RLT buffer using a single 5 mm steel bead in a TissueLyserII (Qiagen) for 5 min at 30 Hz. After this, manufacturer’s instructions were followed and DNA was eluted into 60 µl buffer EB and RNA was eluted into 50 µl RNase-free water. Sequencing libraries were prepared using Nexetra XT kits (Illumina, Cambridge, UK) and sequencing using a Nextseq sequencer (Illumina, Cambridge, UK). Illumina) to generate 2× 150 base paired-end reads.

The raw data were filtered to remove adaptors and low quality reads using the programs fastp version 0.23.4 (Chen et al., 2018) and multiqc v 1.19 (Ewels et al., 2016). The sequences were aligned and the Dipteran host genomes removed in the program Bowtie2 (Langmead and Salzberg, 2012). Sequences were assembled using MEGAHIT (D. Li et al., 2015). Taxonomic classification was carried out using the program Kracken2 (Wood et al., 2019), with the program Bracken (Lu et al., 2017) to estimate species abundance and facilitate removal of poor quality and low abundance sequences. Viral sequence detection was performed using ViralVerify (https://github.com/ablab/viralVerify/tags, last accessed 16 September 2024) and ViralComplete (https://github.com/ablab/viralComplete last accessed 16 September 2024) with sequences obtained from the NCBI viral database (https://www.ncbi.nlm.nih.gov/labs/virus/vssi/ last accessed 16 September 2024). APV Virus sequences identified using these methods were isolated and the identity was confirmed using BLAST+ (Camacho et al., 2009).

### PCR screening of samples

Twenty-four of the flies previously preserved in 70% ethanol were selected from a range of sites across England, across an area where APV is likely to occur in Dunnocks, from sites where APV had not been observed on any bird species by bird ringers, as well as sites from which it had been reported from Dunnocks or other species. Twenty-three flies were from Dunnocks and one from a Great Spotted Woodpecker *Dendrocopos major*.

These flies were not washed, to avoid removing evidence that they might be mechanical vectors. Flies were homogenized in liquid nitrogen prior to DNA extraction, using the DNeasy Blood and Tissue kit (QIAGEN) according to the manufacturer’s instructions. Screening for APV was carried out through amplification of a 578-bp PCR product of the APV 4b core protein gene (*fpv*167) as previously described, using primer sequences 5′-CAGCAGGTGCTAAACAACAA-3′ and 5′-CGGTAGCTTAACGCCGAATA-3′ (Lawson et al., 2012). Amplification was achieved using the high-fidelity Q5 polymerase (NEB) under the following cycling conditions: 98°C (5 min); 40 × [98°C (1 min); 64°C (20 s); 72°C (20 s)]; 72°C (7 min). Following confirmation of a product of the correct size on an agarose gel against a positive control of a DNA sequence from a Dunnock pox lesion at the main site (GenBank Accession number PP756527), APV was confirmed through bidirectional Sanger sequencing (Source BioScience) using the same primers as for amplification.

### Computational analysis

Other analyses were performed and the phenology plotted in R version 4.2.1 (R Development Core Team, 2022) using packages dplyr (Wickham et al., 2023) and lubridate (Grolemund and Wickham, 2011) to process the data. The map was plotted using the packages maps (Becker et al., 2022), mapdata (Brownrigg, 2022) and scales (Wickham and Seidel, 2022).

## Results

In order to investigate the association between APV infection, small Passerine bird species and louse flies, Dunnocks were sampled from sites in England. Two species of louse flies were identified from Dunnocks, *O. avicularia* and *O. fringillina*.

**Figure 1.**
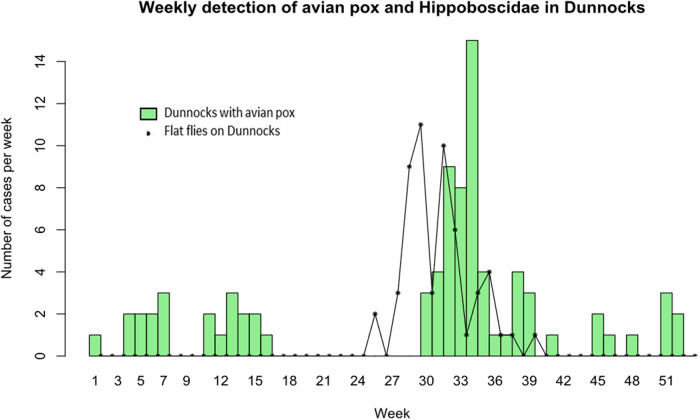
Weekly cases of avian pox in Dunnocks (green bars) and numbers of louse flies (*O. avicularia* and *O. fringillina* combined) found on Dunnocks (black stars and solid lines) for all years combined, from a 16·5 year study in Somerset, UK.

**Figure 2.**
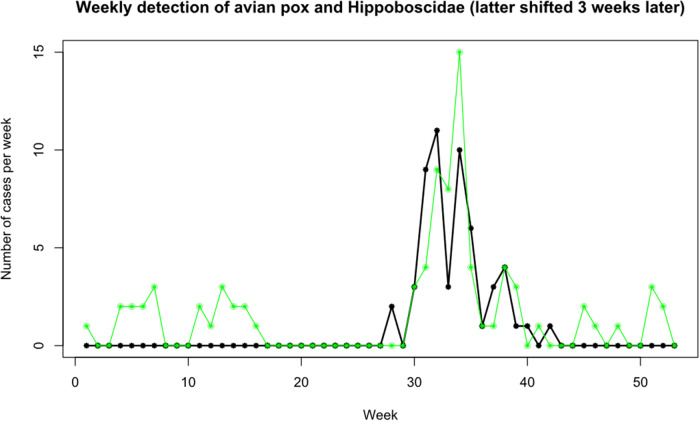
Weekly detection of avian pox in Dunnocks (green dots) and presence of louse flies on Dunnocks (black stars), with the louse flies plotted 3 weeks later than their actual dates, to show the similarity in peaks, otherwise separated by the approximate incubation period of avian pox in Dunnocks.

Figure 1 shows the relationship between Dunnocks with evidence of APV infection and presence of flat flies (both species) and Dunnocks based on cumulative data over the study period. APV infections were reported during the first 16 weeks of the year and then a later surge in infections during weeks 30–36, reaching a peak of 15 cases during week 34, then declining for the remaining weeks of the year. Flat flies were only detected on Dunnocks from week 24 and peaked on week 29 with another peak on week 31. This preceded the peak of APV cases by 2–3 weeks as shown when the louse fly frequency plot is moved to three weeks later (Figure 2).

NGS sequencing and classification with Kracken2 identified five non-overlapping sequences of DNA as APV covering a total of 598 base pairs (Table 1) from an *O. avicularia* taken from a Dunnock with APV at the second site (Table 2). However, the length of sequence detected (<600 base pairs) represents less than 0.2% of the APV genome (comparison with Magpiepox virus 2 complete genome of 298,392 bp, GenBank Acc. No. MW485973). It is not clear if this represents residual presence of virus on the external surface of the louse fly (e.g. contamination of mouthparts following feeding on the Dunnock), despite surface disinfection, or presence within the fly (gut, oral cavity, salivary glands or other internal organs) but at very low levels. No APV RNA was detected.

**Table 1.** Avian pox contig BLAST results from NGS sequencing. This table shows the results for the top blast hit of each of the contigs identified as being avian pox, from the *Ornithomya avicularia* taken from a Dunnock at the second site on the Wirral Peninsula near Liverpool, England

DNA sequence	Top BLAST hit	Identity (%)	Genbank accession number	*E*-value	Query coverage (%)	alignment length (bp)
1	Crowpox virus	100	ON408417.1	7.00E − 25	100	67
2	Canarypox virus	98.9	AY318871.1	2.00E − 36	100	91
3	Canarypox virus	99.21	AY318871.1	4.00E − 56	100	127
4	Canarypox virus	93.62	AY318871.1	2.00E − 50	100	141
5	Canarypox virus	100	AY318871.1	4.00E − 66	100	142

**Table 2.** Louse flies *Ornithomya* spp. Tested for APV. Results of sequencing with details of bird host and site and GenBank accession numbers, for the *Avipoxvirus* 4b core protein gene sequences

Sample number	Date collected	Species	APV detected method	Fly sex	Bird age	Bird with pox?	Species with avian pox at site	Site	Latitude	Longitude	GenBank accession number
Louse fly host Dunnock *Prunella modularis*											
2402	21 Jul 2021	*O. avicularia*	No		Juvenile	No	Dunnocks	Wirral Peninsula, Cheshire	53·31	−3·20	N/a
FF158	15 Jul 2022	*O. fringillina*	No		Juvenile	No	Dunnocks	Minehead, Somerset	51·20	−3·51	N/a
FF26	11 Jul 2020	*O. avicularia*	No		Juvenile	No	Dunnocks	Minehead, Somerset	52·20	−3·51	N/a
FF64	31 Aug 2020	*O. fringillina*	No		Juvenile	No	Dunnocks	Minehead, Somerset	53·20	−3·51	N/a
FF171	01 Aug 2022	*O. avicularia*	No	F	Juvenile	No	Dunnocks	Minehead, Somerset	55·20	−3·49	N/a
3197	26 Jul 2021	*O. avicularia*	No		Juvenile	No	None seen	West Down, Devon	51·14	−4·29	N/a
FF96	11 Jul 2021	*O. avicularia*	No	F	Juvenile	No	None seen	North Hill, Minehead, Somerset	54·21	−3·51	N/a
H003	26 Jun 2022	*O. avicularia*	No	F	Juvenile	No	None seen	Brandon, Suffolk	52·39	0·50	N/a
2959	24 Jun 2022	*O. avicularia*	No	F	Fledgling	No	None seen	Cameley, North Somerset	51·25	−2·57	N/a
2942	01 Sep 2022	*O. fringillina*	No	M	Juvenile	No	None seen	Cameley, North Somerset	51·25	−2·57	N/a
435	12 Jul 2022	*O. avicularia*	No	M	Adult	No	None seen	Little Snoring, Norfolk	52·83	0·82	N/a
X366	25 Jul 2023	*O. avicularia*	No	F	Juvenile	No	None seen	Bridford, Devon	50·61	−3·70	N/a
JT01	14 Jul 2023	*O. avicularia*	NGS	F	Juvenile	Yes	Dunnocks	Wirral Peninsula, Cheshire	53·31	−3·20	N/a
2414	10 Nov 2021	*O. fringillina*	PCR		Juvenile	Yes	Dunnocks	Wirral Peninsula, Cheshire	53·31	−3·20	PQ790037
FF108	02 Aug 2021	*O. fringillina*	PCR	M	Juvenile	No	Dunnocks	Minehead, Somerset	51·20	−3·51	PQ790031
FF122	08 Aug 2022	*O. fringillina*	PCR	M	Juvenile	No	Dunnocks	Minehead, Somerset	51·20	−3·51	PQ790033
FF167	30 Jul 2022	*O. avicularia*	PCR	F	Juvenile	No	Dunnocks	Minehead, Somerset	51·20	−3·51	PQ790032
FF22	07 Aug 2013	*O. fringillina*	PCR		Adult	No	Dunnocks	Minehead, Somerset	51·20	−3·51	PQ790030
1566	16 Jul 2022	*O. fringillina*	PCR	F	Juvenile	No	Great tits	Stanford Reservoir, Northamptonshire	52·41	−1·12	PQ790038
1568	31 Jul 2022	*O. avicularia*	PCR	M	Juvenile	No	Great tits	Corby, Northamptonshire	52·41	−0·83	PQ790036
1569	01 Aug 2022	*O. avicularia*	PCR	F	Adult	No	Great tits	Corby, Northamptonshire	52·41	−0·83	PQ790034
3936	10 Jul 2021	*O. avicularia*	PCR	F	Adult	No	None seen	Near Sandy, Bedfordshire	52·41	−0·38	PQ790039
4960	12 Jul 2022	*O. avicularia*	PCR		Juvenile	No	None seen	Rutland Water, Rutland	52·59	−0·82	PQ790035
X	13 Jul 2022	*O. avicularia*	PCR	M	Juvenile	No	None seen	Rutland Water, Rutland	52·59	−0·82	PQ790040
Fly host Great Spotted Woodpecker *Dendrocopos major*											
7589	18 Sep 2023	*O. avicularia*	PCR	M	Juvenile	No	None seen	West Down, Devon	51·14	−4·29	PQ790029

**Figure 3.**
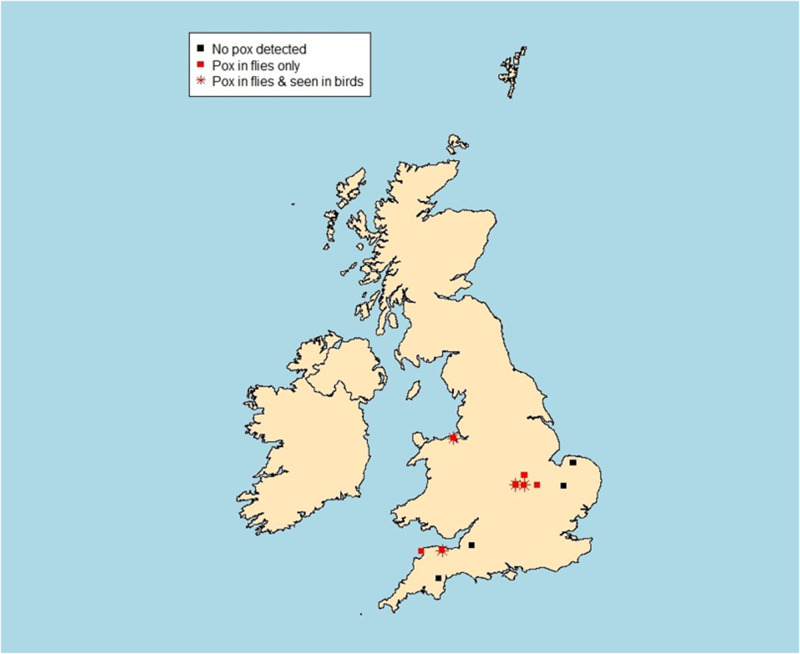
Map of the sites from which flies were tested: black squares – no avian pox detected, in either tested louse flies or observed in birds; red squares – avian pox detected but no birds observed with avian pox; red stars – birds observed with avian pox and flies tested positive.

To more comprehensively test for the presence of APV 24 further samples of louse flies were subjected to PCR-based analysis for APV DNA. Eleven of 23 flies tested from Dunnocks and the one from the Great Spotted Woodpecker (Table 2), were PCR positive for APV. These included one *Ornithomya fringillina* from a Dunnock with APV at the site on Wirral Peninsula near Liverpool. All PCR-derived sequences were identical across the 512 bp where they aligned, and a 100% match to in this high-quality central region of the sequence obtained from an APV lesion on a Dunnock at the main site. These positive flies were from the main study site (Somerset: *n* = 4), two sites (Corby and Stanford Reservoir in Northamptonshire) where APV was occasionally observed in Great Tits *Parus major*, but not in Dunnocks, either during the study or in the previous year (*n* = 3), and three sites (Rutland Water in Rutland, near Sandy in Bedfordshire, and West Down in Devon) where APV had not been observed in any bird species (*n* = 4). The locations of these sites, and those at which no flies tested positive (*n* = 4), are shown in the map (Figure 3).

In total, 13 out of 25 louse flies tested positive for APV, 6/9 *O. fringillina* and 7/16 *O. avicularia*. Six of the flies were recorded as being female, four male and in three the sex was not recorded (Table 2).

None of the Dunnocks without active disease at the time of capture, from which the flies were taken, were later seen to develop APV, although most (7/10) were not recaptured. However, most (7/10) were juveniles at the time of capture and would have been expected to leave their natal areas (the ringing sites) during their postnatal dispersal.

## Discussion

APV DNA was detected in 13 of 25 louse flies, of two species, *O. avicularia* and *O. fringillina*, that were obtained from sites across England. All the 512 base pair sequences obtained from the louse flies by PCR were identical to that obtained from an APV lesion on a Dunnock at the main site, and matched reference sequences from NCBI GenBank. The APV positive flies included one *O. avicularia* and one *O. fringillina* that had been taken from Dunnocks with APV lesions, the rest having been taken from Dunnocks with no signs of disease, and a single fly from an apparently healthy Great Spotted Woodpecker. Field studies showed that peaks in the presence of louse flies on Dunnocks were followed three weeks later by peaks in the prevalence of APV.

The three week interval falls within the estimate of the incubation period of APV in Dunnocks of 13–48 days (Wawman *et al.*, unpublished data). However, phenological comparisons may not be a reliable means of identifying a vector because other vectors’ emergence may be triggered by the same climatic conditions as that of the louse flies. Arthropod vectors are known to down-regulate their hosts’ immune systems which may promote transmission of APV (Wikel, 1999). Moreover, reproduction-associated stress or hormonal induced immunosuppression may also make Dunnocks more susceptible to APV at particular times of the year.

Although conclusive evidence that APV is vectored by Hippoboscidae may be challenging to obtain, the detection of APV DNA associated with louse flies is strong evidence of the potential for a role in transmission. If, as expected from knowledge of its mode of transmission by other vectors, APV is mechanically vectored, evidence of viral replication in the vector, such as Bollinger Bodies (Bollinger, 1873) or the presence of viral mRNA, would not be expected, and indeed RNA was not detected in this study, although only a single specimen taken from a Dunnock with APV was tested, and further testing is needed to confirm this result. Using sequences to compare viral DNA in avian hosts and parasites might confirm that the same strains are circulating in both (Yeo et al., 2019), but would not prove beyond doubt that Hippoboscids are responsible for transmission. In this study, all of the 512 base pair sequences obtained by PCR were identical, and matched that taken from an APV lesion on a Dunnock at the main site, but longer sequences may be necessary to fully establish that they are the same strain of the virus.

Isolating APV DNA from a Hippoboscid taken from a Dunnock that later developed APV, might more strongly implicate Hippoboscids as vectors. However, finding APV DNA in 52% of the louse flies tested, including flies from sites where no APV had been observed in any species of bird, indicates that APV may be circulating in the wild bird population at a far higher rate than expected purely from the observed presence of clinical signs. Lesions were only observed in 8·3% of Dunnocks (Wawman *et al.*, unpublished results) and 7·2% of House Finches *Haemorhous mexicanus* (McGraw et al., 2022), but PCR detected APV in the spleens of 43% of wild birds in Italy (Bertelloni et al., 2022), and 69·2% of a sample of avian species introduced into New Zealand were seropositive (Ha et al., 2013). It would be necessary to use other methods such as invasive sampling techniques to detect the timing of seroconversion in birds in relation to the presence of flies carrying APV, which would be difficult in a wild host population.

Detecting APV in louse flies at sites where no APV has been observed, and at sites where APV has only been observed in species other than Dunnocks, such as at Stanford Reservoir and Corby, where it was only seen occasionally in Great Tits, might suggest that APV is of low virulence in Dunnocks. It is also likely that it exists in an enzootic cycle between different wild bird species and louse flies and other arthropod vectors. The two species of louse flies found to be positive for APV in this study, *O. avicularia* and *O. fringillina* are found on a wide range of bird species (Wawman, Smith and Sheldon, unpublished results) and could be vectoring APV between Dunnocks, Great Tits and other affected species. This hypothesis is supported by the presence of an APV-positive louse fly on a Great Spotted Woodpecker.

Louse flies may have potential as a means of monitoring the potential for vector-transferred disease in wild bird populations. NGS sequencing could be used to determine which pathogens of potential interest are present in louse flies, potentially by pooling specimens to minimise the cost. Pathogen targeted PCR could be used in cases where focal pathogens have already been identified as a potential cause for concern and sequences compared to track different strains of concern. Individual based analyses can be used to quantify the numbers of infected individuals, including those in the prodromal phase of disease and those in which infections remain subclinical, and to determine the geographical areas in which diseases are present. Expanding this approach to keds, could also allow diseases to be tracked in the wild deer population, and in livestock such as horses, sheep and goats in regions where they are frequently infected.

Other parasites have been used to take samples from wild birds, for example, blood sucking bugs, *Dipetalogaster maximus*, contained within dummy eggs have been used avoid stress when taking blood samples from adult nesting birds including Common Terns *Sterna hirundo* for cortisol assays (Arnold et al., 2008), and Eurasian Kestrel *Falco tinnunculus* for counting blood parasites, leucocyte profiles and microsatellite analysis for paternity tests and genetic sexing (Sumasgutnet et al., 2014), and have been shown to be a reliable method in Common Swift *Apus apus* (Bauch et al., 2013). Bat flies (Diptera: Nycteribiidae) which are closely related to the louse flies, and sometimes included as part of the Hippoboscidae, have been used to investigate the presence of *Bartonella* spp. *Polychromophilus* spp. and *Trypanosoma* spp. in bats (Szentiványi et al., 2020).

Using louse flies to screen for the presence of disease in a population would have several advantages compared to using serology or other samples from birds. Firstly, serology and other blood tests require invasive sampling techniques, performed by veterinary surgeons or individuals who have undergone additional training and licensing, which limits the sample size and geographical area over which a study can take place, and adds costs to any project: louse flies can be collected by any bird ringer, or wildlife rehabilitator or member of the public who comes across a sick, injured or dead bird hosting louse flies. Citizen science projects, such as the Mapping the UK’s Flat Flies Project (Wawman, 2025) could allow widespread coverage across a region. Additionally, serology only indicates that a bird has produced an immune response at some point during its life, with no way of determining the timing of the infection, whereas, because of the short life span of Hippoboscids, assuming that transovarial and transstadial transmission can be ruled out, any infection within them is likely to have been acquired in the same season. Adult louse flies have a life span of around three to six months (Hutson, 1984), so APV virions would have to be acquired in the same year. Using PCR-sequencing approaches to detect viral DNA is specific for the disease under investigation, whereas relying on observations of sick birds in the field, or even in the hand by trained bird ringers, risks both mis-diagnoses and a significant underestimate of prevalence of infection.

A major disadvantage of using wild louse flies is that the sampling is likely to be somewhat random in nature, as only birds with ectoparasites that can be caught can be sampled, and there may be differences in the health of birds with and without parasites, especially if co-infections are present. Quantitative results, such as an estimate of disease prevalence will be difficult to obtain. Sick birds may be easier to catch if flying weakly, or be so sick that they remain hidden and are not caught. Ectoparasites may be affected by the presence of avian endoparasites: the louse fly *Olfersia spinifera*, was found to be less likely to switch hosts when infected with *Haemoproteus iwa* (Levin and Parker, 2014) and ectoparasites may aggregate on certain hosts with co-infection. The prevalence of micro-organisms may not be consistent between vectors and their hosts – a study of bats and bat flies showed twice the prevalence of *Trypanosoma* spp. and *Polychromophilus* spp. in hosts compared to their ectoparasites, but a similar prevalence of *Bartonella* spp. in both groups (Szentiványi et al., 2020).

Vertical transmission leading to infection of louse flies might also be a potential issue for some diseases in which it occurs and might explain the higher rate of detection of APV in the louse flies compared to their hosts. *Bartonella schoenbuchensis* is vertically transmitted in the Deer Ked *Lipoptena cervi* (de Bruin et al., 2015), and *Anaplasma ovis* in the Sheep Ked *Melophagus ovinus* (Zhao et al., 2018), but vertical transmission of viral pathogens has not been reported in louse flies. However, vertical transmission of endosymbionts occurs (Duron et al., 2014) and some arthropod viruses are vertically transmitted via both ova and spermatozoa (Longdon and Jiggins, 2012). Some sigmaviruses, a group of RNA viruses found naturally in Diptera, are known from Hippoboscids, such as the Wuhan Louse Fly sigmaviruses (C.-X. Li et al., 2015) and Aksy-Durag Melophagus sigmavirus (Litov et al., 2021). It is possible that compared to the transovarial route, reproduction by adenotrophic viviparity in the Hippoboscidae, could favour vertical transmission, as there is prolonged maternal contact between the female and her offspring, during which time it is fed from a specialised milk gland.

Despite these issues, useful results might be obtained similar to those used in the surveillance used in monitoring other vector borne diseases such as the European network for medical and veterinary entomology (VectorNet) (https://www.ecdc.europa.eu/en/about-us/partnerships-and-networks/disease-and-laboratory-networks/vector-net, last accessed 22 November 2024), which monitors the ranges of mosquitos, ticks, biting sandflies, and midges across Europe, or other studies such as VB-RADAR in the UK, which monitors the flaviviruses, WNV and Usutu Virus in mosquitoes (https://www.vb-radar.com, last accessed 22 November 2024). Using louse flies may have advantages over using other arthropod vectors to monitor disease, because, although louse flies switch hosts (Corbet, 1956), those collected by ringers are taken from a host with a ring bearing a unique identifying code, which allows the bird to be followed throughout its life, potentially giving additional information about the progress of diseases that would not be obtained otherwise.

From the phenological and molecular evidence presented in this paper, it is likely that the *Ornithomya* spp. present in the UK, especially *O. avicularia* and *O. fringillina*, are vectors of APV in Dunnocks. Further research will be required to determine the exact role of louse flies in vectoring APV in Dunnocks, and in other avian species, and how this occurs. Although mechanical transmission would be the expected route, the possibility of vertical transmission facilitating autochthonous disease transmission should be considered.

Louse flies may provide a relatively cheap and non-invasive method of monitoring disease outbreaks (or assessing potential at risk populations) in wild birds, when combined with targeted DNA sequencing for specific pathogens.

## Data Availability

Details of the flies sequenced can be found in Table 1 together with the accession numbers for the APV sequences.
